# Clinical Implications of the General Movement Optimality Score: Beyond the Classes of Rasch Analysis

**DOI:** 10.3390/jcm10051069

**Published:** 2021-03-04

**Authors:** Vanessa Maziero Barbosa, Christa Einspieler, Everett Smith, Arend F. Bos, Giovanni Cioni, Fabrizio Ferrari, Hong Yang, Berndt Urlesberger, Peter B. Marschik, Dajie Zhang

**Affiliations:** 1 UI Health, Occupational and Physical Therapy Department, University of Illinois at Chicago, Chicago, IL 60612, USA; vbarbo1@uic.edu; 2 Research Unit iDN, Interdisciplinary Developmental Neuroscience, Division of Phoniatrics, Medical University of Graz, 8036 Graz, Austria; christa.einspieler@medunigraz.at (C.E.); dajie.marschik@med.uni-goettingen.de (D.Z.); 3 Educational Psychology Department, University of Illinois at Chicago, Chicago, IL 60607, USA; evsmith@uic.edu; 4 Division of Neonatology, Beatrix Children’s Hospital, University Medical Center, University of Groningen, 9713GZ Groningen, The Netherlands; a.f.bos@umcg.nl; 5 IRCCS Fondazione Stella Maris, Department of Developmental Neuroscience, University of Pisa Scientific Director, 56128 Pisa, Italy; gcioni@fsm.unipi.it; 6 Department of Pediatrics and Neonatology, University Hospital Policlinico, University of Modena, 41124 Modena, Italy; ferrarif@unimore.it; 7 Department of Rehabilitation, Children’s Hospital of Fudan University, Shanghai 201102, China; hyang@shmu.edu.cn; 8 Division of Neonatology, Medical University Graz, 8036 Graz, Austria; berndt.urlesberger@medunigraz.at; 9 Child and Adolescent Psychiatry and Psychotherapy, University Medical Center Göttingen, 37075 Göttingen, Germany; 10 Leibniz Science Campus Primate Cognition, 37075 Göttingen, Germany; 11 Karolinska Institutet, Department of Women’s and Children’s Health, Center of Developmental Disorders (KIND), 11330 Stockholm, Sweden

**Keywords:** cerebral palsy, detailed assessment, general movements, preterm infant, neonate, optimality score, Rasch analysis, psychometric properties, clinical implications

## Abstract

This article explores the clinical implications of the three different classes drawn from a Rasch analysis of the general movements optimality scores (GMOS) of 383 infants. Parametric analysis of the class membership examines four variables: age of assessment, brain injury presence, general movement patterns, and 2-year-old outcomes. GMOS separated infants with typical (class 3) from atypical development, and further separated cerebral palsy (class 2) from other neurodevelopmental disorders (class 1). Each class is unique regarding its quantitative and qualitative representations on the four variables. The GMOS has strong psychometric properties and provides a quantitative measure of early motor functions. The GMOS can be confidently used to assist with early diagnosis and predict distinct classes of developmental outcomes, grade motor behaviors, and provide a solid base to study individual general movement developmental trajectories.

## 1. Introduction

The General Movement Assessment (GMA), a categorical analysis of the quality of an infant’s spontaneous general movements, has been established as a systematic, valid, and reliable assessment of the integrity and function of the young nervous system [1,2] and is currently the most recommended assessment for identifying a high risk for cerebral palsy (CP) [3,4].

The GMA is an observational tool based on gestalt pattern recognition of the infant’s spontaneously generated movements from birth to 5-months post term. General movements occur in age-specific patterns. Normally, general movements in preterm and term age comprise the entire body and manifest themselves in a variable sequence of neck, trunk, arm, and leg movements [2]; they wax and wane in intensity, speed, and amplitude. Abnormal general movements at that age are characterized by a lack of variability, especially in the movement sequence [1,2]. Experienced observers achieve high inter-scorer agreements (89–93%) [2].

In addition to the categorical assessment of general movement patterns, a detailed assessment at preterm and term age that examines different components of these movements was first introduced by Ferrari et al. [5], adapted by Einspieler et al. [6], and resulted in the General Movement Optimality Score (GMOS) [7]. The GMOS applies the Prechtl optimality concept [8], resulting in an ordinal semi-quantification of the general movements’ quality, in which neck, trunk, upper, and lower extremities are scored separately, with inter-scorer agreements ranging from 0.69 to 0.82 (Cohen’s Kappa) [7]. In a large group of preterm and term infants, GMOS differentiated not only infants with normal and abnormal movement patterns, but also, within the abnormal classification, poor repertoire and cramped synchronized movements [7]. The relationship between the GMOS and the GMA depended on infants’ age, except for “tremulous movements” items which occurred across infants from all ages with normal and abnormal general movements [7]. The GMOS is utilized both in clinical and research areas. It has demonstrated correlations with the GMA classifications, but poor prediction for the GMA at 3 months corrected age [9]. It is worth noting, however, that significant changes in the movement patterns occur during this period, and likely contribute to the poor prediction. The GMOS has also been used to evaluate short term neurological outcomes of clinical hypoxia [10,11] and of neonatal intervention [12].

Recently, Barbosa et al. [13] explored the GMOS’s psychometric properties using Rasch analysis, a statistical method used in test development. The Rasch probabilistic model [14] considers the measure of the item’s difficulties and a person’s abilities together on the same scale. Rasch transforms ordinal data into linear measure with equal-interval units, called logits, which are used to describe the measures of both individuals and items. Different Rasch analysis models were performed using the GMOS [13] with the Mixed Rasch Model (MRM) presenting the best overall fit, with good to optimum separation indexes and reliability coefficients. This suggests that the GMOS is a reliable interval unidimensional assessment that works differently for three distinct groups of infants that are separated into classes. In MRM, each infant is assigned to one of the classes she or he has the highest probability of belonging to, based on their scoring in each detailed item, so that each class (or group of infants) presents unique functioning rating scale and different item hierarchy. MRM validated the GMOS as a quantitative assessment and proposed improvements, including deleting two items (“tremulous movement”, “upper and lower extremities”) and revising the scoring criteria of 5 others (neck, amplitude, and speed of upper and lower extremities). MRM did not, however, explain who the infants in each class are, making it difficult to establish clinical implications. Therefore, the purpose of this study was to identify the infants who belonged to each class [13], by exploring differences in specific clinical features, including age of assessment, type of brain injury, GMA classification, and clinical outcome at two years of age, and ultimately to understand the essence of the qualitative differences across the classes.

## 2. Experimental Section

Secondary data analyses were performed on previous work by Einspieler et al. [7] and Barbosa et al. [13]. Details of the 383 infants each videotaped once following the standards of the Prechtl’s GMA, the original score sheet, and scoring procedures are published elsewhere [7,13]. A list with the GMOS item names, scoring criteria, and hierarchical items’ structure (i.e., logits by class) is presented in Appendix A (adapted from Barbosa et al. [13]). The ethical boards of all centers involved [7] approved the recording and assessment of spontaneous movements. Data analysis was performed in compliance with protocols approved by the Institutional Review Board of the Medical University of Graz (ethical approval number 27-388ex 14/15; 27-388ex14/15). Institutional Review was deemed not necessary for secondary data analysis by the ethical board of the home institution of the lead authors. Written informed consent was obtained from all participants prior to study.

Frequencies and percentage were used to explain class composition and explore its implications to clinical practice. ANOVAs, cross tabulations, and Chi-square (SPSS version 21; SPSS Inc., Chicago, IL, USA) were used to compare class composition based on age of assessment, type of brain injury, GMA classification, and clinical outcome at two years of age.

Data Availability: The datasets generated and analyzed during the current study are available from the corresponding author on reasonable request.

## 3. Results

Table 1 shows the specific distribution of video recording per gender, age of assessment, brain injury (images/clinical signs: presence of intra ventricular hemorrhage (IVH), periventricular leukomalacia (PVL), or hypoxic ischemic encephalopathy (HIE)/Sarnat classification), GMA classification, and outcome at two years of age; and Table 2 shows the ANOVAs and Chi-square results.

There was a significant relationship between class assignment and age of assessment, both for weeks of age (continuous, *p* < 0.001) as well as age groups (categorical, *p* < 0.001) (i.e., <34 weeks post menstrual age (PMA) vs. ≥34 weeks PMA). Infants in class 2 were older (40.59 weeks) than the ones in class 1 (37.6 weeks) and class 3 (38.73 weeks) (Table 2). The majority of the sample was of older infants. Most of the very preterm and moderate preterm infants (59.4%) belonged to class 1 (27.7% of the class). The late preterm, term, and post-term infants were similarly distributed among classes 1, 2, and 3 (31%, 36.7%, and 32.3% respectively) (Table 1 and Table 2).

The distribution of infants with distinct grades of brain injury (images/clinical sings: normal and grade 1 PVL/IVH/Sarnat classification vs. grades 2, 3, and 4 PVL/IVH/Sarnat classification, combined) was significantly different among the classes, *p* < 0.001. Class 1 had a similar number of infants with normal/grade 1 injury (49.5%) to infants with higher grade injuries (grades 2, 3, and 4) (50.5%). Class 2 had a higher proportion of infants with higher grade injuries (77%) and class 3 was mostly composed of infants with normal/grade 1 injury (78%).

The relationship between class and GMA classification into poor repertoire, normal, cramped synchronized, and chaotic movements differed among the classes: *p* < 0.001. Post hoc analysis demonstrated that all but chaotic movements were differently distributed across the classes, with standard residuals greater than +/− 1.96, *p* = 0.0055.

The association between class and outcome at two years of age (i.e., normal, CP, and neurodevelopmental disorders other than CP) was examined by first combining outcome into typical and atypical among the three classes, which showed a predominant proportion of infants in Class 3 with typical outcome compared to Class 1 and 2, *p* < 0.001. Next, we examined only the atypical infants from classes 1 and 2, which showed that class 1 had the most infants with other neurodevelopmental disorders (67.10%), while class 2 had the most with CP (70.65%), *p* < 0.001.

As there was an overlap between classes 1 and 2, we then further explored the interaction among age of assessment, brain injury, GMA, and outcome separately within these classes with cross-tabulations and chi-square (Table 3) with the intent to further differentiate and understand their uniqueness.

Class 1 was mostly composed of infants with poor repertoire (95.6%), resulting in a nonsignificant interaction between GMA and outcome, *p* = 0.074. Class 2, on the other hand, showed a significant interaction between GMA and outcome, *p* < 0.001. Post hoc analysis showed that the distribution of GMA varied among the outcomes for all but chaotic movements, with standardized residuals greater than +/−1.96, *p* = 0.0055. Although 18.7% of the infants with cramped synchronized movements in class 2 turned out to have other neurodevelopmental disorders, cramped synchronized movements were mostly related to the outcome of CP (81.3%). Moreover, 93.8% of the infants (61/65) in class 2 who were diagnosed with CP had cramped synchronized movements.

We then further explored the characteristics of the infants with poor repertoire within these two classes. The overall proportion of such infants in class 1 (95.6%) was much higher than in class 2 (26.8%). Nevertheless, there was a similar chance (approximately 50%) within both classes that these infants would have other neurodevelopmental disorders (Table 3). In class 1, the other half of the infants was roughly divided between normal outcome and CP; in class 2, only 8.7% of infants with poor repertoire turned out to have CP. Infants with poor repertoire in class 1 had brain lesions similarly distributed between less (48.4%) and more (51.6%) severe; whereas in class 2, there was a higher proportion of infants with more (63.6%) severe injuries. Age also varied between classes 1 and 2 in infants with poor repertoire, being older in class 2 (93.9%) than in class 1 (71%).

No interactions were found between GMA and brain injury, GMA and age of assessment, and brain injury and age of assessment (for all infants), in either class 1 or 2 (Table 3). The relationship between brain injury and outcome was significant for all infants in both classes. Class 1 presented a higher frequency of other neurodevelopmental disorders, both in the lower and higher injury groups (*p* = 0.043). Normal outcome, however, was more frequent in the lower grade injuries, and CP in the higher-grade injuries. Class 2 had a higher proportion of infants with CP in both injury groups (42.1% and 73% in low and high injury, respectively, *p* = 0.045). Normal outcome and other neurodevelopmental disorders were more frequent in the lower grade injuries. Nevertheless, when interaction between brain injury and outcome was considered given the GMA classification, an interaction was no longer significant in either class 1, *p* = 0.072, or class 2, *p* = 0.452.

Finally, we explored the relationship between brain injury and outcome distribution given the age groups (<34 and ≥34 weeks PMA). Class 1 showed a significant relationship for the younger infants, *p* = 0.013, but not for the older ones, *p* = 0.267. Younger infants with lower grade injuries had more normal and other neurodevelopmental disorders, whereas younger infants with higher grade injuries had a higher incidence of CP. Contrarily, in class 2, a significant relationship was found for the older infants, *p* = 0.031, but not for the younger ones, *p* = 1.000. Older infants had higher grade injuries (62/80, 77.5%) and a higher frequency of CP (45/62, 72.5%).

## 4. Discussion

The validity of the GMOS as a quantitative assessment to describe different aspects of the quality of general movements was recently demonstrated [13]: MRM indicated the existence of three distinguishable groups of infants (i.e., classes) for whom the GMOS needs to be considered separately as the items’ difficulties and infants’ abilities are class dependent. Therefore, describing who the infants were in each class allowed for understanding the specific relationship between individual GMOS items and specific clinical features, including outcomes. This information could be very useful in a clinical setting, helping to introduce therapy to the infants who need it the most.

### 4.1. Class Formation

Exploring the commonalities and differences of infants in each class clarified their clinical relevance. The overall class composition is described next (also Table 1 and Table 2). Class 1 was the most diverse class, with 27.7% younger infants (<34 weeks PMA), mixed grades of brain injury, and mostly infants with poor repertoire classifications in GMs. Half of these infants were later diagnosed with other neurodevelopmental disorders (the other half divided between normal and having CP) at two years of age. Class 2 was mostly older infants (≥34 weeks PMA) (95.1%), higher levels of brain injury, mostly cramped synchronized GMs, and infants later diagnosed with CP. Class 3 was mostly older infants (83.7%), no to grade 1 brain injury, normal GMs, and later diagnosed as presenting a normal outcome.

While infants of all ages might have poor GMOS performance, age of assessment was a factor influencing class formation. The interaction between class membership, presence of brain injury, GMA classifications, and outcome at two years of age was also statistically significant and helped guide the interpretation of how GMOS items work differently for each group of infants. The GMOS clearly differentiated infants with typical/normal outcome from those with atypical development (i.e., CP and other neurodevelopmental disorders). Class 3, with more infants with normal features, explains the results in Barbosa et al. [13], which showed for this class most favorable movement qualities (i.e., higher GMOS total raw and logit scores, and more individual items scored in the highest score categories). Classes 1 and 2, contrarily, presented mixed groups of infants with some overlap between them. Examining the interaction among age of assessment, brain injury, GMA, and outcome separately within each of these classes (Table 3) allowed us to further appreciate their qualitative differences and demonstrated their unique relationship to atypical development.

Class 1’s composition explains the normal-like distribution of GMOS scores we previously reported [13]. It also supports the literature in which a large proportion of preterm infants have transient abnormal general movements [15,16] (categorized as poor repertoire in particular), that can either normalize or deteriorate within the first few months of life and result in different outcomes [1,2,7,17,18] (i.e., normal, CP, attention deficit/hyperactivity disorder -ADHD, cognitive impairment, developmental coordination disorder, autism). An important clinical implication for class 1 is that these infants need to be closely monitored to differentiate between transient and enduring abnormality to guide intervention as necessary.

Class 2’s composition explains the least optimal quality of movements reported for this class [13]. This is also in agreement with previously reported lower total GMOS scores for post term infants [7,9] and supports that outcome tends to be worse if infants do not normalize their movement patterns sooner [1]. This is not surprising, given most infants in this class had cramped synchronized movements, which appear at approximately 35–36 weeks PMA [6,7] and concur with lack of fluency and variability of GMOS movement components [7]. It also confirms the predictive value of cramped synchronized movements with the outcome of CP [19,20]. Given the highest reliability for this class in our previous study [13], its composition also suggests that the GMOS best separates infants with CP from the others (Table 2).

Besides, while supporting previous research in which infants with normal GMA tend to have normal outcome and infants with cramped synchronized GMA tend to have CP [7,20], our results provided further information on the development of infants with poor repertoire GMA. Half of the infants with poor repertoire tend to present other neurodevelopmental disorders at two years of age, regardless of which class they belonged to. The other half, however, developed differently, pending on which class they belonged to. The combination of poor repertoire with brain injury and age of assessment further contributed to clarifying the trajectory of infants with poor repertoire, separating them and providing preliminary information to predict their outcome. Having poor repertoire GMA concomitant with being younger was more associated with class 1, with higher probability of presenting neurodevelopmental disorders other than CP, regardless of brain injury level. On the other hand, having poor repertoire, being older, and presenting higher levels of lesion was more related to class 2, with a higher chance of having CP.

### 4.2. Relationship between GMOS Scores (Total and Item Performance) and Clinical Populations

These results allow us to interpret how infants with different clinical features and outcomes scored on the GMOS total score and on each item. Detailed comparison of item difficulties across classes is a particularly informative procedure about the qualitative differences among the classes. Specifically, in the GMOS, this helps understanding the differences in the motor performance by different groups of infants. The focus of this comparisons is on the items which are relatively more difficult for one group but easier for the others. We encourage the reader to revisit Barbosa et al. [13] and further explore specific items’ scores per class, noticing the items’ total score (logits) are all different among the classes, with the easiest items located at the bottom of the table for each group of infants (Appendix A).

A practical example of this is the item “space: lower extremities”. This item difficulty order is similar between infants with normal outcome (class 3) and those with other neurodevelopmental disorders (class 1), being 19th and 17th, respectively. The quality of the use of space varies between these classes, however, with infants with normal outcome scoring mostly as showing variability in space, and infants with other neurodevelopmental disorders scoring mostly with limited use of space [13]. For infants with CP (class 2), on the other hand, the item “space: lower extremities” was placed in a higher difficulty order (11th) than other items, although they also tended to have limited use of space and had a similar item location (i.e., logit) as infants in class 1. This suggests that while having similar logits, it is much harder for an infant later diagnosed with CP to have variable use of space than either infants later diagnosed with other neurodevelopmental disorders or infants with normal outcome. Some clinical implications of this could be to observe the easiness of progression (in repeated tests) in the lower extremities use of space, which can help differentiate those that might develop CP (slower gain in motor control) from those who will go on to have other neurodevelopmental disorders. It might also help in planning intervention, by facilitating the behaviors beginning to emerge, possibly using a passive range of motion to provide variability to self-initiated movement while also offering environmental affordances that stimulate active movement (i.e., placing toys at different locations in relationship to the infant’s body to stimulate kicking).

### 4.3. A Word of Caution

All analyses were based on secondary and group data. New studies with prospective data and a wide range of the infant population are needed to investigate the modified scoring system and its application in enhancing the understanding of general movements’ subtleties and prediction of outcome. To continue the research of the GMOS, now knowing it is a working metric scale, future steps would be to (1) examine potential GMOS cut-off scores to discriminate infants with different outcomes, and to (2) identify who would improve or deteriorate in their components of movement over time by studying the GMOS developmental trajectories. This would allow one to further predict long-term neurodevelopmental outcome and to track subtle changes in the quality of movement resulting from intervention (rehabilitation and/or pharmacological interventions). Another interesting question to explore in the future is if the combination of total GMOS scores (e.g., above or below the median) with GMA classification (e.g., poor repertoire) can assist with outcome prediction.

## 5. Conclusions

Our previous study demonstrated (using Rasch analyses/MRM) that the GMOS is a reliable unidimensional assessment with functioning rating scales and different item hierarchy for qualitatively differentiating infants, grouping them into three classes [13]. In the present study, we identified the clinical features of each class. Combining the results of both studies provides valuable information and supports the psychometric validity of the GMOS as a solid and reliable quantitative assessment that works differently for three clinical meaningful groups of infants, providing further content and discriminative validity evidence for the GMOS quantitative properties. The modified GMOS separated infants with typical (class 3) from atypical development (classes 1 and 2), and then further separated CP (class 2) from other neurodevelopmental disorders (class 1) based on the combination of their specific clinical features. While further predictive studies are needed, we recommend using the GMOS to evaluate infant motor performance in more detail and assist in referring an infant sooner for targeted intervention, during times of greater brain plasticity, when a greater impact of intervention may be seen.

## Figures and Tables

**Table 1:** Class detail and sample description by class membership.

	TOTAL = 383 *N* (%)	Class 1 (*n* = 137)	Class 2 (*n* = 123)	Class 3 (*n* = 123)
**Gender**				
Male	232 (60.6%)	97	80	55
Female	151 (39.4%)	48	66	37
**Age of Assessment**				
Very Preterm Period (26^+0^ to 31^+6^ weeks PMA)	35 (9.1%)	**21**	2	12
Moderate Preterm Period (32^+0^ to 33^+6^ weeks PMA)	29 (7.6%)	**17**	4	8
Late Preterm Period (34^+0^ to 36^+6^ weeks PMA)	71 (18.5%)	25	15	**31**
Term (37^+0^ to 41^+6^ weeks PMA)	87 (22.7%)	21	**45**	21
Post-term Period (42^+0^ to 45 weeks PMA)	161(42.1%)	**53**	**57**	**51**
**Brain Injury (Images and Clinical Signs)**				
Normal	99 (25.8%)	30	6	**63**
PVL grade 1 or IVH grade I, or Sarnat I	40 (10.4%)	**18**	14	8
PVL grade 2 or IVH grade II, or Sarnat II	94 (24.5%)	33	**42**	19
PVL grade 3 or IVH grade III and IV, or Sarnat III	42 (11.1%)	16	**25**	1
Missing data	108 (28.2%)	40	36	32
**GMA Classification**				
Normal	116 (30.3)	2	7	**107**
Poor Repertoire	179 (46.7)	**131**	33	15
Cramped Synchronized	83 (21.7)	3	**80**	0
Chaotic	5 (1.3)	1	3	1
**Outcome at 2 years of age**				
Normal	147 (38.4%)	27	16	**104**
Cerebral Palsy	92 (24%)	25	**65**	2
Other Neurodevelopmental Disorders	85 (22.2%)	**51**	27	7
Unknown *	59 (15.4%)	34	15	10

PMA, post menstrual age; PVL, periventricular leukomalacia; IVH, intra ventricular hemorrhage; * Infants had not yet reached 2-year for a reliable outcome assessment; GMA, General Movement Assessment; Bold, indicates highest number per class membership.

**Table 2:** ANOVAs and Chi-square examining the General Movement Optimality Score (GMOS) logit mean scores and class membership based on different clinical variables.

	Class 1	Class 2	Class 3
Summary of our previous results ◊			
Reliability Index—ANOVA	0.862	0.955	0.748
Reliability Index—Andrich	0.840	0.953	0.664
Category scoring probability	Mostly category 1	Mostly category 0	Category 2 for all items
♦GMOS Mean Raw Score (SD)	20.681 (5.205)	15.175 (9.858)	33.153 (5.157)
♦GMOS Mean Logits (SD)	0.441 (1.217)	−1.361 (3.171)	3.086 (1.408)
Class vs. GMOS Logits	F = 210.148 DF = 2/380 *p* < 0.001		
Post hoc—Tukey Test			
Class 1		<0.001	<0.001
Class 2			<0.001
Summary of current results			
**Class vs. Age**			
***Continuous***	F = 12.167 DF = 2/380 *p* < 0.001		
Mean age	37.6 weeks	40.59 weeks	38.73 weeks
Post hoc—Tukey Test			
Class 1		*p* < 0.001	*p* = 0.150
Class 2			*p* = 0.008
***Categorical***	X^2^ (2, *N* = 383), 24.36, *p* < 0.000		
<34 weeks PMA = 64	27.7%	4.9%	16.3%
≥34 weeks PMA = 319	72.3%	95.1%	83.7%
**Class vs. Brain Injury (Images and Clinical Signs)**	X^2^ (2, *N* = 275) = 53.957, *p* < 0.001		
NL/Level 1 = 139	49.5%	23%	78%
Levels 2, 3, 4 = 136	50.5%	77%	22%
**Class vs. GMA**	X^2^ (6, *N* = 383) = 464.607, *p* < 0.001		
Post hoc—Tukey Test	Standard Residuals > +/−1.96, *p* = 0.0055		
NL = 116	1.5%	5.7%	87%
PR = 179	95.6%	26.8%	12.2%
CS = 83	2.2%	65%	0%
*CH = 5	0.7%	2.4%	0.8%
**Class vs. 2-year Outcome**	X^2^ (2, *N*= 324) = 155.212, *p* < 0.001		
Typical (NL) =147	26.2%	14.8%	92%
Atypical (CP + Other) = 177	73.8%	85.2%	8%
***Atypical vs. class 1 and 2***	*X^2^* (1, *N* = 168) = 23.855, *p* < 0.001		
CP = 90	32.9%	70.7%	
Other = 78	67.1%	29.3%	

◊, reference [13]; PMA, post menstrual age; NL, normal or no brain injury; Level 1 = PVL grade 1 or IVH grade I, or Sarnat I; Level 2, 3, 4 = (PVL grade 2 or IVH grade II, or Sarnat II) + (PVL grade 3 or IVH grade III and IV, or Sarnat III); NL, normal; PR, poor repertoire; CS, cramped synchronized; CH, chaotic; CP, cerebral palsy; Other, neurodevelopmental disorder other than cerebral palsy. ♦, Mean raw and logit scores were from Barbosa et al., 2020. *CH = chaotic was not differently distributed across the classes

**Table 3:** Cross-tabulation and Chi-square examining the interaction between GMA, Outcome, Brain Injury, and age of assessment separately within class 1 and class 2.

**Class 1—*N* = 137**								
**GMA (*n* = 137)**		**Outcome (*n* = 103)**			**Brain Injury (*n* = 97)**		**Age of Assessment (*n* = 137)**	
		**NL**	**CP**	**Other**	**NL + Level 1**	**Level 2, 3, and 4**	**<34 weeks**	**≥34 weeks**
NL	2 (1.5%)			1 (100%)	2 (100%)			2 (100%)
PR	131 (95.6%)	27 (27.6%)	22 (22.4%)	49 (50%)	45 (48.4%)	48 (51.6%)	38 (29%)	93 (71%)
CS	3 (2.2%)		3 (100%)			1 (100%)		3 (100%)
*CH	1 (0.7%)			1 (100%)	1 (100%)			1 (100%)
Total	137 (100%)	27 (26.2%)	25 (24.3%)	51 (49.5%)	48 (49.5%)	49 (50.5%)	38 (27.7%)	99 (72.3%)
**Chi-squares within Class 1**								
GMA by Outcome					X^2^ (6, *N* = 103), 11.515 *p* = 0.074			
GMA by Brain Injury					X^2^ (3, *N* = 97), 4.087 *p* = 0.252			
GMA by Age of assessment					X^2^ (3, *N* = 137), 2.409 *p* = 0.494			
Brain Injury by Outcome					X^2^ (2, *N* = 82), 6.273 ***p* = 0.043**			
Brain Injury by Outcome given GMA					X^2^ (2, *N* = 82), 5.271 *p* = 0.072			
Brain Injury by Age of assessment					X^2^ (1, *N* = 82), 2.556 *p* = 0.110			
Brain Injury by Outcome given age <34 weeks PMA					X^2^ (2, *N* = 24), 8.640 ***p* = 0.013**			
Brain Injury by Outcome given age ≥34 weeks PMA					X^2^ (2, *N* = 58), 2.638 *p* = 0.267			
**Class 2—*N* = 123**								
**GMA (*n* = 123)**		**Outcome (*n* = 108)**			**Brain Injury (*n* = 87)**		**Age of Assessment (*N* = 123)**	
		**NL**	**CP**	**Other**	**NL + Grade 1**	**Grades 2, 3, and 4**	**<34 weeks**	**≥34 weeks**
NL	7 (5.7%)	7 (100%)			2 (50%)	2 (50%)		7 (100%)
PR	33 (26.8%)	9 (39.1%)	2 (8.7%)	12 (52.2%)	8 (36.4%)	14 (63.6%)	2 (6.1%)	31 (93.9%)
CS	80 (65%)		61 (81.3%)	14 (18.7%)	10 (16.9%)	49 (83.1%)	4 (5%)	76 (95%)
*CH	3 (2.4%)		2 (66.7%)	1 (33.3%)		2 (100%)		3 (100%)
Total	123 (100%)	16 (14.8%)	65 (60.2%)	27 (25%)	20 (23%)	67 (77%)	6 (4.9%)	117 (95.1%)
**Chi-squares within Class 2**								
GMA By Outcome					X^2^ (6, *N* = 108), 83.678 *p* < 0.001			
Post hoc:					Standardized residual +/−1.96			
GMA					Outcome			
NL					Normal vs. CP Normal vs. Other		*p* = 0.0055 *p* = 0.0055	
PR					Normal vs. CP Normal vs. Other CP vs. Other		*p* = 0.0055 *p* = 0.0055 *p* = 0.0055	
CS					Normal vs. CP CP vs. Other		*p* = 0.0055 *p* = 0.0055	
GMA by Brain Injury					X^2^ (3, *N* = 87), 5.684 *p* = 0.128			
GMA by Age of assessment					X^2^ (3, *N* = 123), 0.781 *p* = 0.854			
Brain Injury by Outcome					X^2^ (2, *N* = 82), 6.207 ***p* = 0.045**			
Brain Injury by Age of assessment					X^2^ (1, *N* = 82), 0.829 *p* = 0.363			
Brain Injury by Outcome given GMA					X^2^ (2, *N* = 82), 1.587 *p* = 0.452			
Brain Injury by Outcome given age <34 weeks PMA					X^2^ (1, *N* = 2), 0.000 *p* = 1.000			
Brain Injury by Outcome given age ≥34 weeks PMA					X^2^ (2, *N* = 80), 6.965 ***p* = 0.031**			

NL, normal; CP, cerebral palsy; Other, other neurodevelopmental disorders; Level 1 = PVL grade 1 or IVH grade I, or Sarnat I; Level 2, 3, 4 = (PVL grade 2 or IVH grade II, or Sarnat II) + (PVL grade 3 or IVH grade III and IV, or Sarnat III); PVL, periventricular leukomalacia; IVH, intra ventricular hemorrhage; PR, poor repertoire; CS, cramped synchronized; CH, chaotic. * CH= chaotic was not differently distributed across the classes.

## Data Availability

The datasets generated and analyzed during the current study are available from the corresponding author on reasonable request.

## Appendix A

**Table A1:** Mixed Rasch Model: Item Measure and Location by Classes; Analysis with item “tremulous movements”, upper and lower extremities deleted.

		Class 1	Class 2	Class 3
General Movements Optimality Scores (GMOS) Item List and Scoring		Item Difficulty by Class		
Item Number—Name/(Abbreviation)	Scoring Criteria *(italics = original scoring)*	Item Number—Abbreviation/Logit (SE)		
1—Sequence (Seque)	2—variable 1—monotonous and/or broken 0—synchronized /disorganized	13—LEspeed 1.005 (0.140)	18—LEoffset 4.500 (0.270)	5—UEspeed 1.450 (0.186)
2—Neck (Neck)	2—variably involved in the sequence 1—moves isolatedly 0—does not move at all *2 = involved* *1 = hardly or not involved*	5—UEspeed 1.002 (0.139)	10—UEoffset 3.264 (0.239)	8—UEdisrot 1.334 (0.191)
3—Trunk (Trunk)	2—fluent and elegant rotations 1—just a few rotations 0—almost no rotation	18—LEoffset 0.790 (0.421)	1—Seque 1.939 (0.259)	4—UEampli 1.269 (0.197)
4—Upper Extremities Amplitude (UEampli)	2—variable, full range 1—monotonous 0—predominantly small/large *2 = variable, full range* *1 = predominantly small/ large/monotonous*	1—Seque 0.790 (0.371)	17—LEonset 1.740 (0.251)	6—UEspace 1.124 (0.198)
5—Upper Extremities Speed (UEspeed)	2—variable 1 –monotonous 0—predominantly slow/fast *2 = variable* *1 = fast/slow/one speed*	3—Trunk 0.764 (0.185)	19—LEcramp 1.547 (0.263)	3—Trunk 1.112 (0.199)
6—Upper Extremities Space (UEspace)	2—full space variably used 1—limited 0—in one plane only	15—Eproxrot 0.732 (0.187)	11—UEcramp 0.507 (0.223)	13—LEspeed 0.885 (0.233)
7—Upper Extremities Proximal Rotation (UEproxrot)	2—present, fluent, elegant 1—just a few rotations 0—almost no rotations	16—LEdisrot 0.467 (0.181)	9—UEonset 0.380 (0.226)	2—Neck 0.778 (0.210)
8—Upper Extremities Distal Rotation (UEdisrot)	2—present, fluent, elegant 1—just a few rotations 0—almost no rotations	4—UEampli 0.310 (0.142)	15—LEproxrot 0.299 (0.231)	7—UEproxrot 0.763 (0.214)
9—Upper Extremities Onset (UEonset)	2—smooth and fluctuating 1—minimal fluctuations 0—predominantly abrupt	12—LEampli 0.301 (0.156)	3—Trunk 0.024 (0.215)	11—UEcramp 0.515 (0.212)
10—Upper Extremities Offset (UEoffset)	2—smooth and fluctuating 1—minimal fluctuations 0—predominantly sudden release	17—LEonset 0.142 (0.292)	16—LEdisrot −0.137 (0.214)	9—UEonset 0.459 (0.229)
11—Upper Extremities Cramped Components (UEcramp)	2—absent 1—occasionally present 0—predominantly present	7—UEproxrot 0.009 (0.174)	14—LEspace −0.802 (0.198)	10—UEoffset 0.343 (0.221)
12—Lower Extremities Amplitude (LEampli)	2—variable, full range 1—monotonous 0—predominantly small/large *2 = variable, full range* *1 = predominantly small/ large/monotonous*	9—UEonset −0.218 (0.216)	7—UEproxrot −0.836 (0.202)	12—LEampli −0.177 (0.341)
13—Lower Extremities Speed (LEspeed)	2—variable 1 –monotonous 0—predominantly slow/fast *2 = variable* *1 = fast/slow/one speed*	6—UEspace −0.224 (0.188)	8—UEdisrot −1.057 (0.201)	17—LEonset −0.182 (0.319)
14—Lower Extremities Space (LEspace)	2—full space variably used 1—limited 0—in one plane only	8—UEdisrot −0.238 (0.169)	13—LEspeed −1.659 (0.218)	15 -LEproxrot −0.443 (0.281)
15—Lower Extremities Proximal Rotation (LEproxrot)	2—present, fluent, elegant 1—just a few rotations 0—almost no rotations	10—UEoffset −0.420 (0.272)	6—UEspace −1.731 (0.215)	1—Seque −0.460 (0.278)
16—Lower Extremities Distal Rotation (LEdistrot)	2—present, fluent, elegant 1—just a few rotations 0—almost no rotations	2—Neck −0.814 (0.185)	12—LEampli −1.831 (0.225)	16 LEdisrot −0.463 (0.283)
17—Lower Extremities Onset (LEonset)	2—smooth and fluctuating 1—minimal fluctuations 0—predominantly abrupt	14—LEspace −0.941 (0.216)	5—UEspeed −1.958 (0.202)	19 -LEcramp −1.759 (0.224)
18—Lower Extremities Offset (LEoffset)	2—smooth and fluctuating 1—minimal fluctuations 0—predominantly sudden release	19—Ecramp −0.980 (0.166)	4—UEampli −2.240 (0.200)	18—LEoffset −2.550 (0.302)
19—Lower Extremities Cramped Components (LEcramp)	2—absent 1—occasionally present 0—predominantly present	11—Ecramp −2.480 (0.202)	2—Neck −3.074 (0.241)	14—LEspace −3.996 (0.549)

Item difficulty hierarchy based on logits: in each class, items listed from the hardest (on top of table) to the easiest (bottom of table); higher logit values indicate higher item difficulty. Item logit = Rasch Measure; SE, Standard Error.
